# Evaluation of Different Subperiosteal Implant Thicknesses on Mechanical Strength and Stress on Bone by Finite Element Analysis

**DOI:** 10.7150/ijms.91620

**Published:** 2024-06-17

**Authors:** Abdulsamet Kundakcioglu, Mustafa Ayhan

**Affiliations:** Istanbul University Faculty of Dental Medicine Department of Oral and Maxillofacial Surgery, Istanbul, Turkey

**Keywords:** Custom Made Implant, Subperiosteal Implant, Bone Resorption

## Abstract

Implants have always been within the interest of both clinicians and material scientists due to their places in reconstructive and prosthetics surgery. Excessive bone loss or resorption in some patients makes it difficult to design and manufacture the implants that bear the necessary loads to carry the final prosthetics.

With this study; we tried to determine the minimum material thickness of the subperiosteal implants that can withstand the physiological forces. We have created a digital average bone structure based on actual patient data and designed different subperiosteal implants with 1, 1.5, and 2mm material thicknesses (M1, M2, M3) for this digital model.

The designed implant models are subjected to 250 Newtons (N) of force, and the implant and bone are tested for the stress they are exposed to, the pressure they transmit to, and their mechanical strength with Finite Element Analysis with the physical parameters boot for the implant material and human bone.

Results show us that under specific design parameters and thicknesses, the 1mm thickness design failed due to exceeding the yield stress limit of 415MPa with a 495,44MPa value. The thinnest implant showed plastic deformation and transmitted excessive forces, which may cause bone resorption due to residual stress.

We determined that thinner subperiosteal implants down to 1.5mm that have the necessary material parameters for function and tissue support can be designed and manufactured with current technologies.

## 1. Introduction

Adentia associated with severe bone resorption is one of the most challenging conditions to treat in maxillofacial surgery. The significant loss of bone in patients' jaws makes implant-supported prostheses either very difficult or impossible, as well as using removable prostheses extremely uncomfortable. Since endosseous implants need sufficient bone tissue to surround them, grafting operations are required before implant applications in cases of bone resorption.

Various surgical techniques, such as onlay iliac grafting and zygoma implants, have been described to treat this condition. 1-3 Although the reconstruction of the jaw bones with iliac grafting is a successful method, it has some disadvantages, such as a more extended treatment period, the impossibility of using a temporary prosthesis, the fact that a second surgical site is involved, and the patient's temporary walking difficulty.

In cases of reconstruction with zygoma implants, zygomatic implants have their own specific problems. We can list these problems as sinus-related infections, soft tissue problems, prosthetic issues, and implant failures.^4^, ^5^ On the other hand, digital developments in computer-aided design and production software in oral and maxillofacial surgery offer various solutions to the difficulties in subperiosteal implant applications.

The aperture piriformis and zygomatic buttress regions of the upper jaw, which are the areas where subperiosteal implants would be placed, remain intact enough to carry occlusal loads and are not affected by bone resorption, even in patients with severe bone atrophy.^6^ Today, with the well-known laser sintering technique currently used in clinical settings, it is possible to make customized titanium subperiosteal implants that are highly compatible with the bone.^7^ With these implants, which can be designed specifically for the patient, fixed prosthetic treatments can be designed by taking support from the appropriate parts of the upper jaw with mini-screws.^8^ Since the implants are placed subperiosteally, they become susceptible to infection in cases of gingival recession; thus, it is crucial to design the implants as thin as possible, various studies are in literature achieve this problem.^9^

We aimed to determine the minimum thickness that can be used for the designs clinically without compromising the necessary material strength or causing any loads to the supporting bone tissue by evaluating the behavior of implants with 3 different thicknesses (1mm, 1.5mm, 2mm) under the predetermined normal occlusal force using the finite element analysis method.

## 2. Materials and Methods

### Study Type and Location

This experimental laboratory study was carried out in Istanbul University Faculty of Dentistry, Department of Oral and Maxillofacial Surgery (Turkey) with the partnership of BioTecnica Engineering, Medical Company (Turkey). Ethical approval of the study is issued by İstanbul University Local Ethics Committee with the number of 2023/12 Rev-1.

### Patient and Data Selection

Between 2018 and 2021, 49 patients who applied to our clinic for implant treatment but were found to have insufficient bone tissue for conventional implant treatment in clinical and radiographic examinations were examined for custom subperiosteal implant treatment. In further examinations, 33 patients with uncontrolled comorbid factors, bisphosphonate use, cleft lip and palate history, or smoking were evaluated as unfavorable regarding a subperiosteal implant. All patients are over 60 years old or above. Despite the indication, four patients refused to be treated voluntarily. Subperiosteal implant treatment was applied to 12 admitted patients at different times. Pre-op and post-op radiographic information of 11 patients who underwent the application was transferred to digital media. One patient was not included in the study because post-op follow-ups could not be performed for reasons unrelated to the study.

During the period spanning from 2018 to 2021, our clinic assessed 49 patients who sought implant treatment but were deemed ineligible due to insufficient bone tissue based on clinical and radiographic evaluations. Subsequently, these patients were considered for custom subperiosteal implant treatment. Upon further examination, 33 individuals were identified as unsuitable candidates due to uncontrolled comorbidities, bisphosphonate usage, history of cleft lip and palate, or smoking habits, making them ineligible for subperiosteal implant placement. All eligible candidates were aged over 60 years. However, four patients declined treatment voluntarily despite meeting the criteria. Eventually, subperiosteal implant treatment was administered to 12 patients at various time points. Radiographic data, both pre-operative and post-operative, were digitally documented for 11 of these patients. One patient was excluded from the study due to the inability to conduct post-operative follow-ups, unrelated to the research.

### Numerical Data Processing

CT (Computed Tomography) scans for 11 patients as volumetric binary files (VBF), first grouped as one file cluster. Model to Model Distance Module of 3DSlicer (Open Source) was performed on the file cluster, and a distance map between 11 models was computed. This distance map creates corresponding point-to-point distance tables with anatomically selected points. Using a principal-component analysis module with computation of the mean group selected a mean value is determined for the group. After the mean value determination for the group, this data is used to generate a template model with the Shape Variation Analyzer module of the 3DSlicer (Open Source). The Shape Population module visualizes the generated model, and the resulting 3D model is used for all the following subperiosteal implant designs. This re-generated 3D model is based on all the mean values of the 11 patient**'**s data and contains all anatomically relevant points.

### Construction of Geometric Models

**Figure 1:**
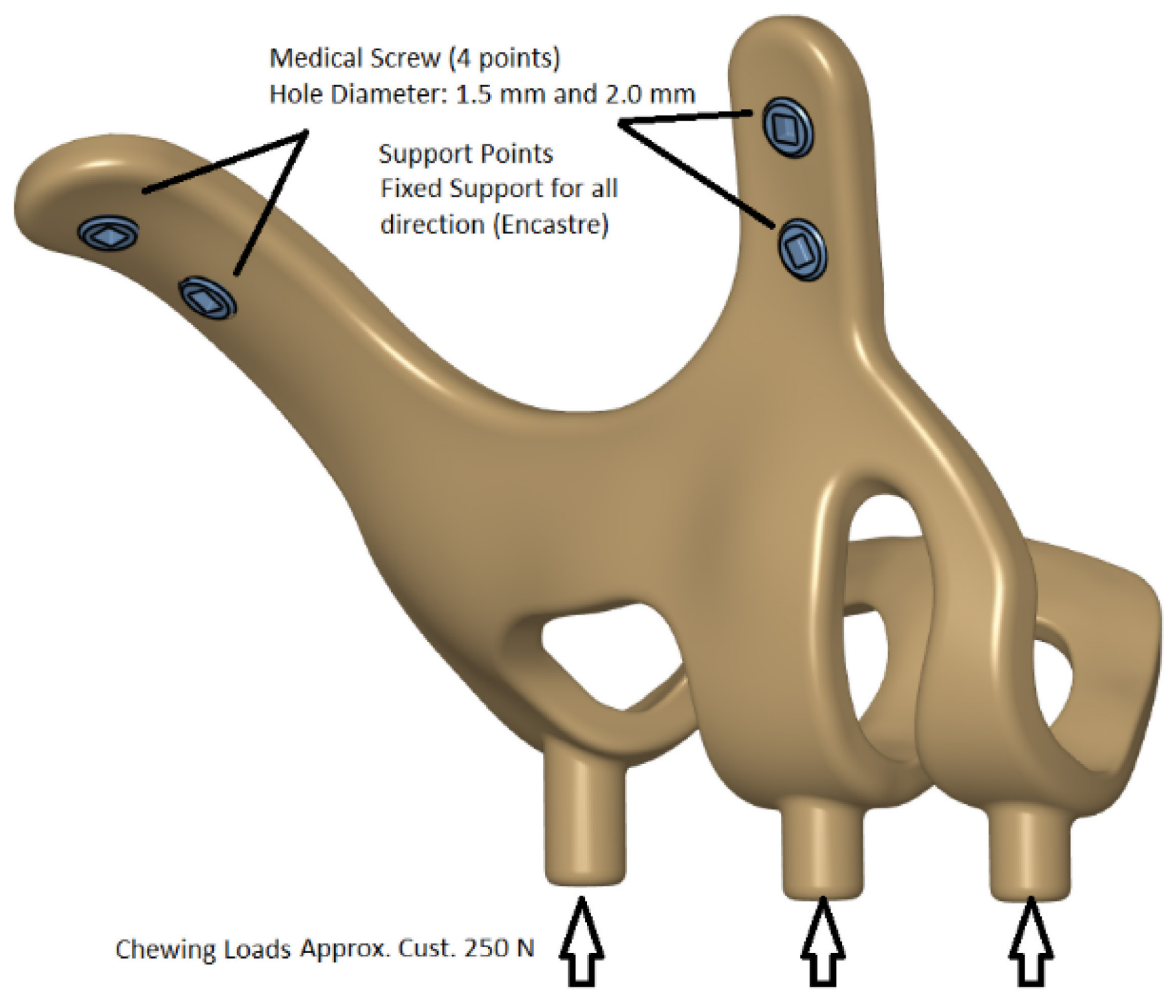
Implant models 3D CAD geometry and boundary conditions.

**Figure 2:**
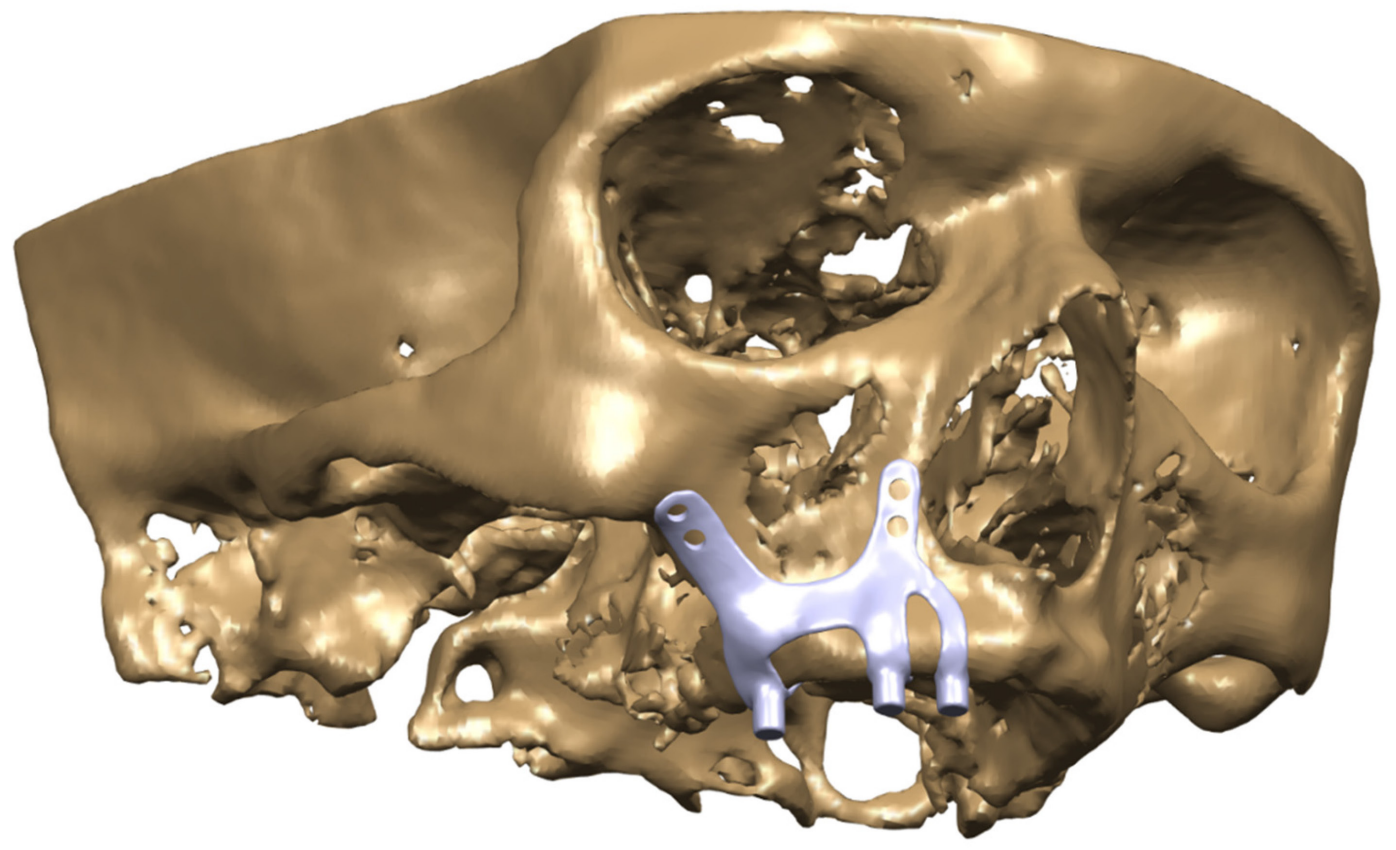
CAD model image of the implant integrated into the maxilla bone.

**Figure 3:**
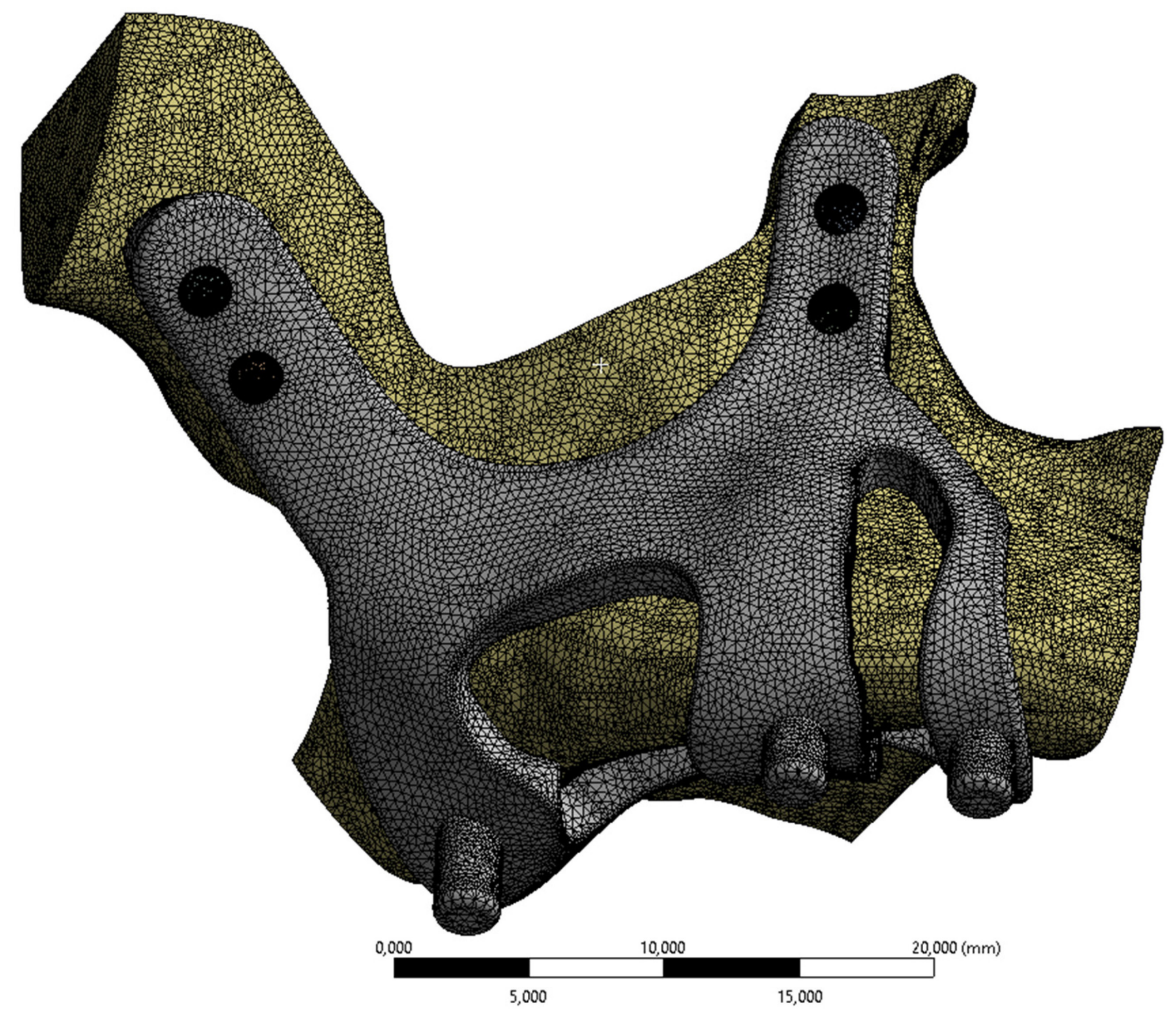
Implant and maxilla bone geometry mesh model image.

Models in Stereolithography (STL) format designed with BioTechnica® medical engineering company were imported into CAD (Computer Aided Design) software. The reverse engineering module of the CAD software was used to convert the 3D models taken as point clouds into solid models. CATIA software was used for CAD applications. The 3D solid model required to analyze the implant geometry was obtained (Figure 1,2,3). Minimizing the deviation between the obtained 3D model and the point cloud data is imperative. For this, deviation analysis has been made for all surfaces (Deviation Analysis) obtained by region definitions. The amount of deviation was determined as 0.05 mm.

FEA (Finite Element Analysis) was used to determine the stress distribution, overall, consisting of bone and implant. The 3D solid model obtained with CAD was transferred to FEA, and a 3D solution mesh was created with Mesh Generation. The finite element method is a numerical method that allows us to obtain information about the structure by dividing the structure into a finite number of small elements and solving a finite number of equations instead of an infinite number of equations. For this reason, the established solution network is vital for the calculation result. ANSYS software was used for FEA applications. An adaptive mesh was applied in the finite element model that was established. The mesh sizes used in the parts forming the whole, the modulus of elasticity on bone and implant, and the poison ratios of the materials used are given in Table 1 and Table 2. Fixation considered immobile and osseointegration of implant frame were denied. The solution matrix is calculated as a tetrahedron mesh type and as a parabolic element. The mesh size was calculated as 0.5mm.

### Load Conditions and Stress Analysis

In our study, stress distribution and analysis were performed for 3 different models under a vertical load of 250 newtons. The stress distribution and effects on the implants were calculated with the Von-Misses yield criterion, but since the bone is not in a homogeneous structure and von Misses can only be used in homogeneous structures, the stress analysis on the bone was calculated according to the Piola-Kirchhoff stress tensors theorems. Although the calculations of implant and bone stress values were done separately, stress measurements were made based on bone-implant contact points to obtain meaningful results.

Residual stress is the internal stresses that occur in the material at the point where the response of the internal structure of the material to this force at the molecular level is equal to the external force as a result of a force applied to a homogeneous material in a static position. These internal stresses remain below the modulus of plasticity, where the material does not lose its plastic properties until a certain point, and when the external force is removed, the internal stresses formed in the material disappear, and the material returns to its original state. If the residual stress in the material exceeds the yield strength of the material, irreversible plastic defects occur in the material.

In our study, the reliability coefficient was determined as 2 to evaluate the plastic deformation to be detected in the material, and according to this safety coefficient, the von-Misses stress value on the implant should be less than half the yield strength of the material to avoid plastic deformation. The yield strength of the Ti6Al4V material is 830 MPa and values below 415 MPa, and it is assumed that it does not undergo plastic deformation.

Vairo G 10 it is accepted that the physiological residual stress limit at which the bone will not be damaged is 170 Mpa for cortical bones in compression and 100 Mpa when tensile forces are applied. Accordingly, when calculated according to the constant of 2, our safety factor in our study, the residual stress in the bone should be below 50 MPa. If these values are exceeded, irreversible collagen destruction and resorption in the bone are possible.

## 3. Results

**Figure 4:**
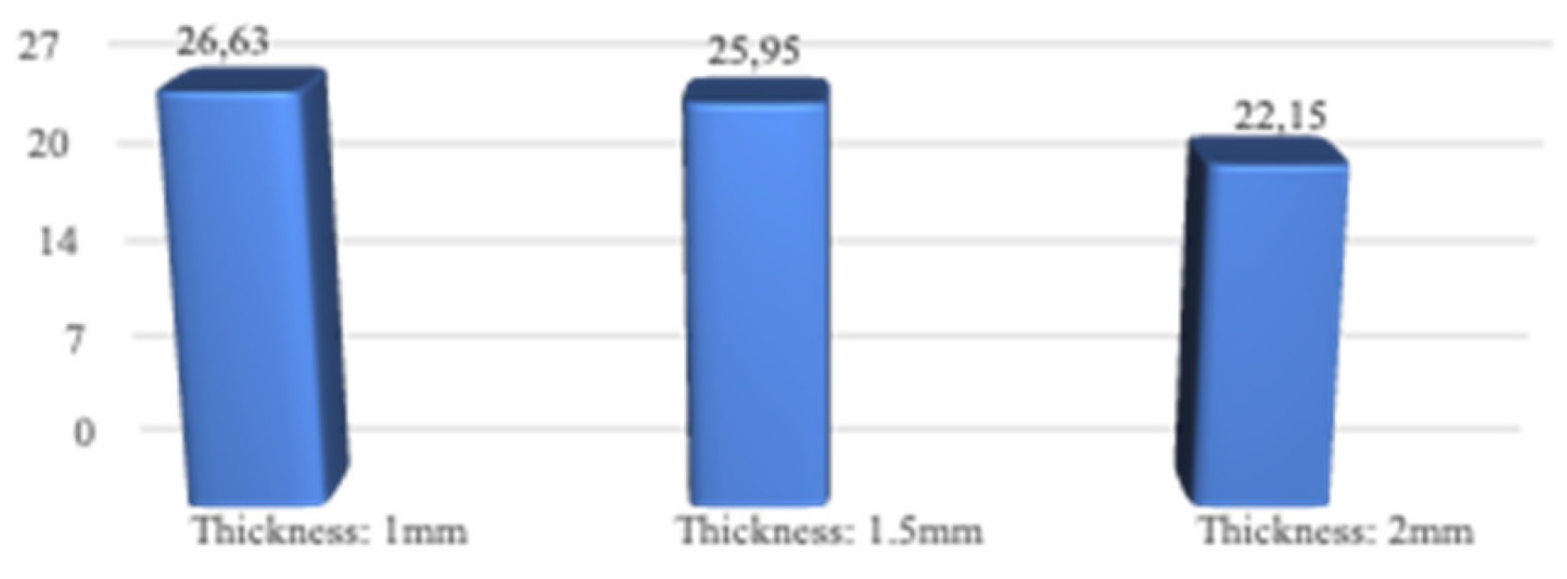
Residual stress changes on bone according to different thicknesses.

**Figure 5:**
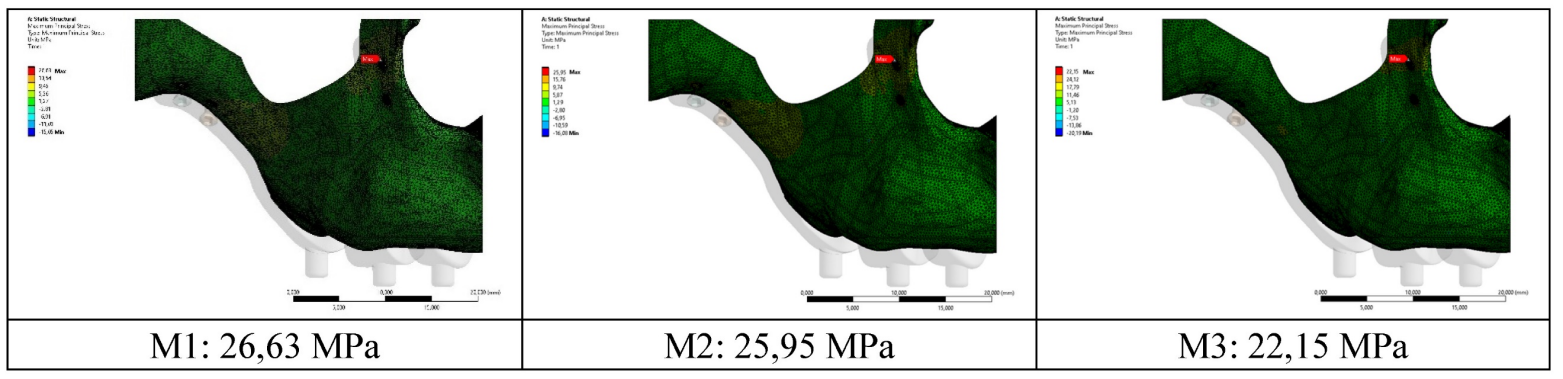
Changes in maximum residual stresses on bone with implants with different thickness.

When the highest residual stress values formed in the bone as a result of vertical loading were examined in all models, it was observed that the lowest was 22.15 MPa in the M3 model with a thickness of 2.0 mm. The highest value was 26.63 Mpa in the M1 model with 1.0 mm thickness (Figure 4,5). The thinness of the implant harmed residual stresses since the pressure on the bone created a displacement force. In addition, this effect will increase the stress on the implant.

**Figure 6:**
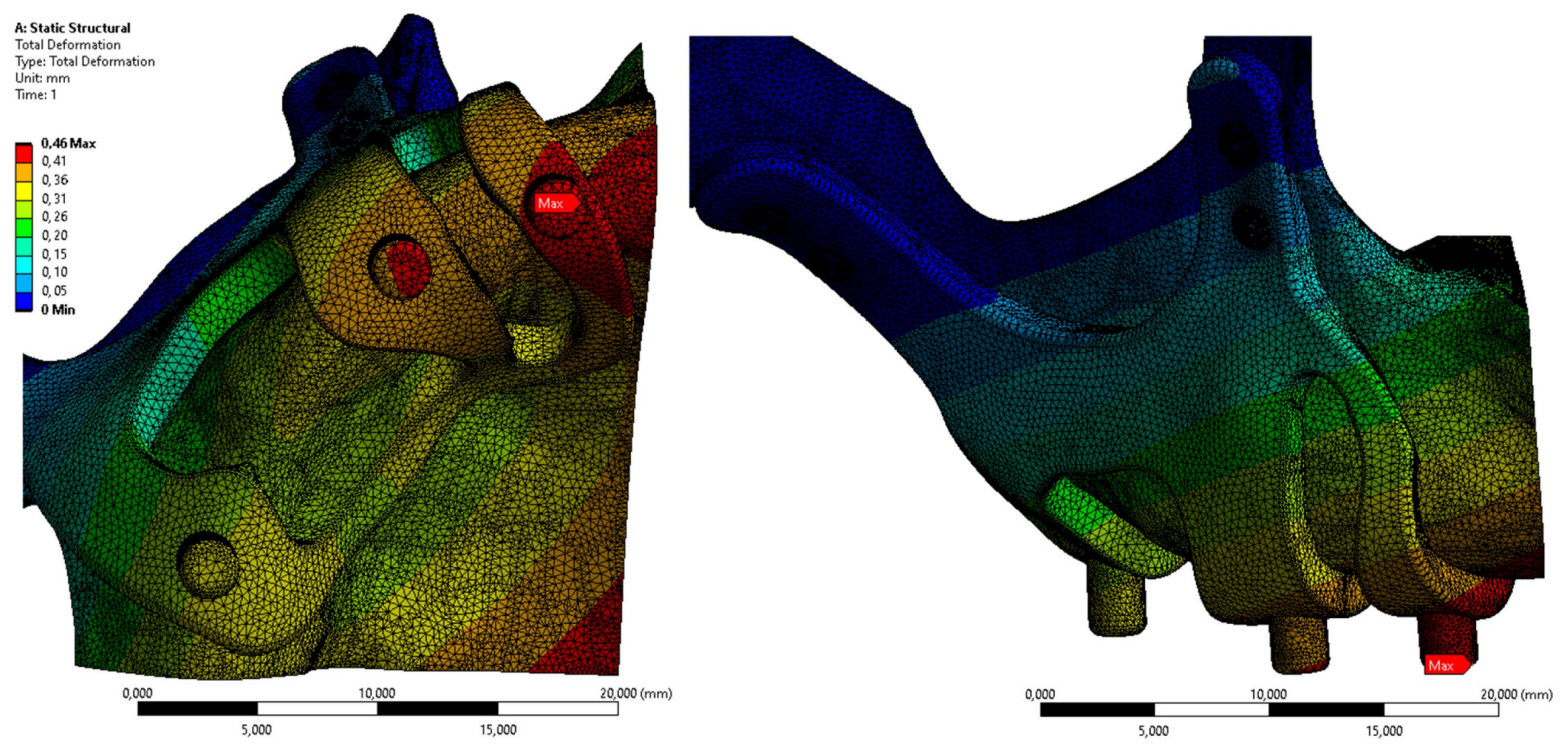
Displacement values on the bone with M3 configuration.

In Figure 6, the displacement value on the M3 model, which has the least residual stress on the bone, is given. When the axial and total displacement values were examined, the highest displacement values on the implants were 1.44 mm in the M1 configuration. The lowest displacement value is 0.46 mm in the M3 implant formed with a 2 mm thickness (Figure 6).

**Figure 7:**
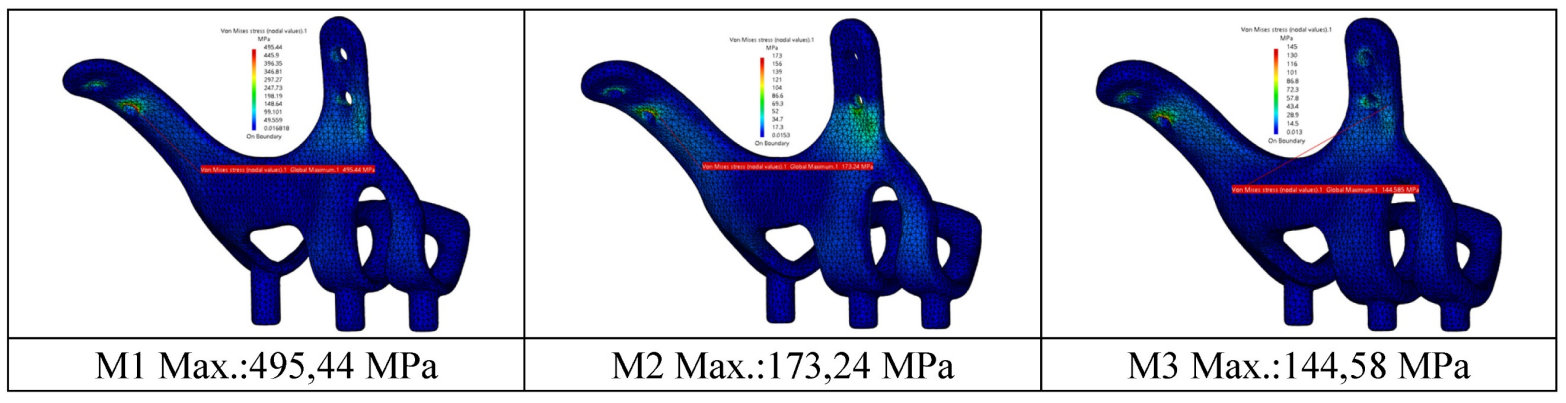
View of von Mises stress values on implants in different thickness.

**Figure 8:**
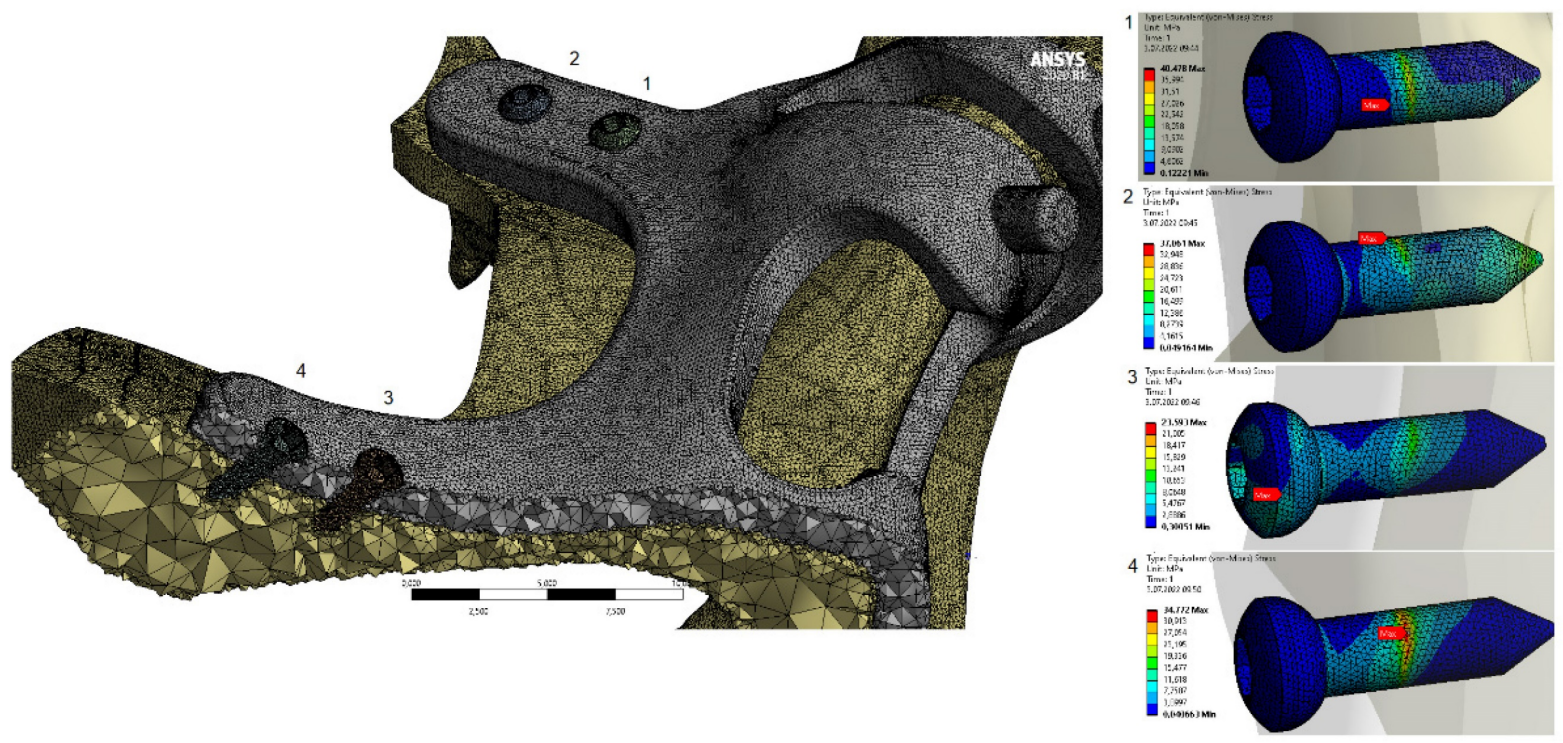
General view of von-Mises stress values on fasteners with M3 configuration.

When the von Mises stress results in the implant are examined, it is seen that the highest stresses occur at the screw connection interfaces in all models (Figure 7). It was determined that the lowest stresses occurred in the M3 model with a thickness of 2 mm, and the highest stresses occurred in the M1 model with a thickness of 1 mm. This is due to the high accumulation of stresses at the thin-walled connection interfaces. On the other hand, with the decrease of the implant thickness, the stress accumulated connection interface also changed. As seen in Figure 8, the highest stresses occurred around the H2 hole in the M1-M2 geometries, while in the M3 geometry, they occurred around the H1 hole (Figure 8).

It is seen that the stresses occurring in implants with thicknesses of 1.5 mm and 2.0 mm under chewing loads are lower than the yield strength of the material. For M2-M3 models, plastic deformation will not occur under static load. It has been observed that the stresses are distributed more homogeneously on the body with increased material thickness and decreased by 3 times compared to the highest stress value. It has been determined that the highest stress regions change with the increase in the thickness of the implant. However, the increase in thickness also increases the weight of the material at a significant rate. The lightest and heaviest implant weights are 2.50 g (M1) and 4.80 g (M3), respectively.

## 4. Discussion

In the discussion, we highlight the historical context of subperiosteal implants dating back to the 1940s and their decline before 3D production techniques due to clinical challenges.^11^-^14^

Endosseous implants replaced them with the advent of osteointegration, particularly for edentulous patients requiring fixed prosthetic treatment.^15^,^16^

However, severe bone deficiency often necessitates bone augmentation techniques like iliac bone grafting (IBG) or zygoma implants, each with its own set of complications. 17-19 Bone grafting using cranial bone yields better long-term results in terms of resorption compared to iliac bone grafting. However, both techniques exhibit similarly high rates of post-operative complications.^20^

These bone augmentation techniques may be painful and require longer treatment periods. To avoid such bone augmenting procedures, zygoma implants have been suggested as a treatment alternative. However, this method has various problems, including sinus infections, eye-related complications, and prosthesis attachment points at undesirable points. 21,22

Subperiosteal implants have been used for the fixed prosthetic rehabilitation of partially edentulous patients with severe bone loss.^8^ Therefore, we performed our study on a model with bone resorption in the form of Cawood-Howell type 4. The use of subperiosteal implants, first applied by Dahl in the 40s, has increased in recent years with the development of imaging and production techniques. Today, they are digitally designed and produced by a 3D laser sintering method that provides fast and effective treatment for patients with severe bone deficiencies and started to become popular again.^8^,^23^

Modern production techniques ensure compatibility with bone, with stability enhanced through screw placement and fixation under local anesthesia.^24^ In our study, the implants were designed to be fixed with 4 screws in each half jaw so that the implants had stable results; although they were made without screws in the past, they can be easily applied under local anesthesia.

One of the significant disadvantages of subperiosteal implants is that they are in direct contact with the periosteum. This can cause a gingival recession or exposure or even implant infection. Patient selection should be considered to avoid such complications, and factors such as diabetes and smoking should be avoided.^25^ It is thought that one of the reasons for this complication is the metal thickness of the implants.^26^ To prevent openings in the gingiva, we evaluated the stresses and pressures on the implants and prosthesis under the chewing force using the finite element analysis method. Our research determines how much the implant thickness can be reduced. We aimed to achieve a design that is resistant enough to withstand chewing forces and thin enough to prevent gingival recessions.

To ensure that the implants can be applied under local anesthesia, they must be placed in the zygomatic prominence and apertura piriformis area without requiring much flap reflection. Subperiosteal implants are designed as two separate parts, as they will be easier to place in the operation and sufficient to elevate the soft tissue in a smaller area surgically.^8^,^23^ Many different designs have been proposed for the subperiosteal implant in the literature. In the old designs, some implants are designed to get support from the apertura piriformis, zygomatic prominence, and palatal dome, also spread over the entire occlusal surface. This way, it aims that the implant will receive maximum support from the bone and be resistant to lateral movements.^27^

With 3D imaging and production methods, designs have been made virtually. One of the essential advantages of this method in the literature is that we can determine the regions where the screws will be placed to increase stabilization.^28^ In this way, the volume of the implants is ensured to be smaller and stress can be reduced.^29^,^30^

In the first trials of this method, the implants are produced as resin in the 3D printer and cast as metal in the dental laboratory. In the laser sintering method, which comes into use later, the production of the implant is made directly by producing the metal in the 3D printer. This production technique is vital in terms of preventing the mistakes that will be encountered in the laboratory stages.^31^

In the literature review, although there is information about the required thickness of the old type subperiosteal implants produced on the model, such a study for the implants produced by the laser sintering method has yet to be observed.^32^

In our study, the stresses and movements on the implant and bone are measured by applying a force of 250N to the implant. The strength of the implants against this force is evaluated. Three subperiosteal implants (1mm, 1.5mm, 2mm) with the same design are modeled in different thicknesses. Residual and Von Mises forces on the implants under 250N occlusal force, displacement amounts, and residual stresses in the bone are measured. For the stresses on the implants, the equivalent value of 415 MPa is exceeded by the 1 mm thick implant, which shows us that the 1 mm thick implant will undergo plastic deformation under occlusal forces. It is predicted that plastic deformation problems may be encountered in implant geometries with a thickness of 1.0 mm. Implants with 1.5 mm and 2 mm thicknesses are safe in terms of plastic deformation as they remain below 415MPa.

When we look at the movements that occur under the force in our study, it is seen that the most extensive movement occurs in the occlusal region of the implant, which is 1mm thick, and the amount of movement is determined as 1.4mm. A minor movement occurs in the 2mm-thick implant and the same area. The movement value is 0.42mm. Minimal movements are acceptable in intraoral applications, but a movement of 1.4 mm cannot be considered minimal. This amount of movement can cause stress accumulation on the implant, fractures in the prosthesis, and resorption in the bone on which it is placed.^33^-^35^

Stress on the bone is one of the causes of bone resorption, and using a proper thickness subperiosteal implant can aid in the avoidance of this resorption.^36^

In our study, 1mm implants are seen as the implants that cause the most stress on the bone, and the slightest stress occurs with the 2 mm-thick implants. Considering the von Mises stresses on the implants, the highest stress is seen at 1 mm and the least stress at 2 mm. When we look at the stresses on the implants in our study, it is seen that the maximum stress areas of the implants of different thicknesses are different. When we look at the stresses occurring at the bone connection points of the implants, it is seen that the screw hole in the apertura piriformis inferior to the 2mm-thick implant is under maximum stress. In 1.5 and 1 mm implants, the screw at the inferior part of the zygomatic prominence is observed to be exposed to maximum tension. As we know from surgical experience, the bone quality of the zygomatic prominence region is higher than the aperture piriformis. For this reason, it should be preferred that the area where maximum tension is desired should be the zygoma region.

When we look at the stress areas on the implant, it is seen that there are no significant stresses around the abutment connection. This result is meaningful for us because we can narrow the abutment implant connecting arms, which is essential to prevent soft tissue retraction that may be seen around the implant. In addition, the thin and narrow connection arms allow the soft tissue to cover the implant faster and thicker after the operation. When we look at the location and values of the stress, the connection between the anterior and posterior screw protrusions of the implants can be narrowed. In this way, the metal area on the bone is reduced, the risk of exposure is reduced, and the implant becomes lighter than respectively.

## 5. Conclusions

With the development of material science in dentistry and the ability to use digital technology in many disciplines, the most crucial advantage of personalized implants produced using the laser sintering method is that they facilitate treatment and shorten the process.^12^,^33^ In this method, thanks to the digital and clinical records taken from the patient before the surgery, a temporary prosthesis can be made with a light and durable material, which can be fixed with screws to the abutments placed on the subperiosteal implant, can be applied to the patient in the same day, thus enabling the patient to start using the prosthesis as soon as possible. In the 1-year follow-up results published by Van den Borre and colleagues, they reported that the treatment results of subperiosteal implant patients met the patients' expectations.^37^ These results suggest that custom subperiosteal implants are an essential alternative for reconstruction patients with severe bone atrophy, especially compared to other treatment options such as iliac bone augmentation and zygomatic implants. However, since laser-sintering custom subperiosteal implant use is a relatively recent advancement, the number of studies on this subject and the clinical experience of physicians is limited. In our study, we tried to determine the minimum implant thickness for clinical use, and according to the results we obtained, the 1.5 mm thick implant showed sufficient strength. Of course, this result, which we obtained with the finite element method, should be evaluated clinically with long-term studies.

## Figures and Tables

**Table 1:** Mechanical properties of Ti6Al4V material and Bone (D2)

Materials	Tensile Strength (MPa)	Modulus of Elasticity (GPa)	Yield Strength (MPa)	Poisson Ratio	Density(kg/m^3^)	Hardness (Hv)
Ti6A14V	960-1270	100-120	830	0.33	4430	320-370
Bone (D2)	120	15	120	0.25	1908	33-43

**Table 2:** Number of elements used in finite element analysis

Model	Mass(gr)	Volume(mm^3^)	Mesh size (mm)	Number of Nodes	Number of Elements
M1 - T:1.0mm	2.52	565	0.5mm	105392	62235
M2 - T:1.5mm	3.66	822		124187	75454
M3 - T:2.0mm	4.80	1076		142020	87879
